# Theranostic Attributes of Acoustic Cluster Therapy and Its Use for Enhancing the Effectiveness of Liposomal Doxorubicin Treatment of Human Triple Negative Breast Cancer in Mice

**DOI:** 10.3389/fphar.2020.00075

**Published:** 2020-02-20

**Authors:** Nigel Bush, Andrew Healey, Anant Shah, Gary Box, Vladimir Kirkin, Sue Eccles, Per Christian Sontum, Spiros Kotopoulis, Svein Kvåle, Annemieke van Wamel, Catharina de Lange Davies, Jeffrey Bamber

**Affiliations:** ^1^ Joint Department of Physics, Institute of Cancer Research, London, United Kingdom; ^2^ Phoenix Solutions AS, Oslo, Norway; ^3^ Department of Physics, CRUK Cancer Therapeutics Unit, The Institute of Cancer Research, London, United Kingdom; ^4^ Norwegian University of Science and Technology (NTNU), Trondheim, Norway

**Keywords:** acoustic cluster therapy, microbubbles, ultrasound, drug delivery, doxorubicin, breast cancer

## Abstract

**Introduction:**

Acoustic cluster therapy (ACT) comprises co-administration of a formulation containing microbubble/microdroplet clusters (PS101), together with a regular medicinal drug (*e.g.*, a chemotherapeutic) and local ultrasound (US) insonation of the targeted pathological tissue (*e.g.*, the tumor). PS101 is confined to the vascular compartment and, when the clusters are exposed to regular diagnostic imaging US fields, the microdroplets undergo a phase-shift to produce bubbles with a median diameter of 22 µm when unconstrained by the capillary wall. *In vivo* these bubbles transiently lodge in the tumor’s microvasculature. Low frequency ultrasound (300 kHz) at a low mechanical index (MI = 0.15) is then applied to drive oscillations of the deposited ACT bubbles to induce a range of biomechanical effects that locally enhance extravasation, distribution, and uptake of the co-administered drug, significantly increasing its therapeutic efficacy.

**Methods:**

In this study we investigated the therapeutic efficacy of ACT with liposomal doxorubicin for the treatment of triple negative breast cancer using orthotopic human tumor xenografts (MDA-MB-231-H.luc) in athymic mice (ICR-NCr-Foxn1^nu^). Doxil^®^ (6 mg/kg, *i.v.*) was administered at days 0 and 21, each time immediately followed by three sequential ACT (20 ml/kg PS101) treatment procedures (n = 7–10). B-mode and nonlinear ultrasound images acquired during the activation phase were correlated to the therapeutic efficacy.

**Results:**

Results show that combination with ACT induces a strong increase in the therapeutic efficacy of Doxil^®^, with 63% of animals in complete, stable remission at end of study, *vs*. 10% for Doxil^®^ alone (p < 0.02). A significant positive correlation (p < 0.004) was found between B-mode contrast enhancement during ACT activation and therapy response. These observations indicate that ACT may also be used as a theranostic agent and that ultrasound contrast enhancement during or before ACT treatment may be employed as a biomarker of therapeutic response during clinical use.

## Introduction

A prerequisite for successful therapy with a medicinal drug is that the active substance reaches its target pathology and that toxicity to healthy tissue and non-targeted organs is limited. Once a drug is administrated systemically, the mononuclear phagocyte system, the vascular endothelium, the disrupted tumor blood flow, the tumor stroma, endosomal escape, and drug efflux pumps are a few among a multitude of other biological barriers that severely restrict its effective delivery from the vascular compartment into the tissue of the targeted pathology (Nizzero et al., 2018). In effect: for a number of drugs, the current, passive transvascular delivery paradigm is inefficient, insufficient, and often results in therapeutic agents failing to reach effective local concentrations due to poor tumor penetration. In combination with low therapeutic indexes, increasing the dosages is not a viable strategy due to serious and widespread adverse effects, overall severely limiting the clinical utility of a range of potent drugs.

While the lack of sufficient extravasation of drug to the targeted pathology is an issue over the range of medicinal therapeutic sectors, this is in particular the case within medicinal treatment of cancers. Regular chemotherapeutics and a range of more novel immune therapies induce severe side effects at partially effective doses and typically, these medicinal regimes are not terminated because the cancer is eradicated, but because the body cannot tolerate more treatment. The outcome is palliative benefit or life prolongation instead of a cure. In conditions such as triple negative breast cancer (TNBC) treated with standard of care chemotherapy, this is unfortunately the case. Specifically, TNBC is a cancer that lacks the expression of estrogen, progesterone, and human epidermal growth factor 2 receptors and is strongly correlated with a poorer outcome when compared to other breast cancer subtypes (Bianchini et al., 2016). This is primarily due to the inherently aggressive clinical behavior, lack of recognized molecular targets for therapy, and heterogenous response to therapy. In 2018, there were estimated to be over 2 million new cases of breast cancer making it the second largest cancer occurrence worldwide (Bray et al., 2018). While significant progress has been made for treatment of breast cancer, there is still a 20% overall mortality ratio at over 620,000 patients per year. The survival impact of TNBC is even worse and commonly referred to as the “kiss of death” (Jitariu et al., 2017) as it is unfortunately deadly in most cases. Specifically, patients diagnosed with TBNC have a mortality incidence from 40 to 50% (Foulkes et al., 2010; Gonçalves et al., 2018) This clearly indicates an important need to improve the therapeutic efficacy for the treatment of breast cancer and even more so of TNBC.

To resolve this fundamental problem, over the past decades, a wide range of concepts to improve the pathology-specific drug uptake (targeted drug delivery) have been explored (Devarajan and Jain, 2015). Within oncology, numerous drug carrier concepts, e.g., liposomes, micelles, dendrimers, and nanoparticles have been employed, either to passively make use of the enhanced permeability and retention (EPR) effect (Maeda et al., 2000), or in combination with surface ligands that actively promote accumulation in tumor tissue through biochemical affinity to specifically expressed target groups. However, even though huge resources have been spent on finding functional concepts for targeted drug delivery over the last two decades, and despite promising pre-clinical results for several of these, there has been very limited transition to drug products and clinical practice. In truth, the objective remains essentially unresolved in current standard-of-care medicinal therapy.

In recent years, several concepts for ultrasound (US) mediated drug delivery have been investigated, some with quite encouraging results (Tsutsui et al., 2004; Martin and Dayton, 2013; Unga and Hashida, 2014). Most of these concepts explore the use of regular US contrast microbubbles such as SonoVue^®^, Optison™, or Definity^®^, either loaded with or co-injected with various active ingredients. Insonation of the target pathology containing microbubbles in vascular compartments leads to a variety of biomechanical effects that enhance extravasation and distribution of drug molecules to target tissue (Kooiman et al., 2014; Lentacker et al., 2014) Co-injection of Gemcitabine and SonoVue^®^, with localized US insonation for a hypothesized enhanced drug uptake and therapeutic effect during treatment of pancreatic cancer (PDAC) has been explored in clinical trials with encouraging results (Dimcevski et al., 2016). A similar approach is being investigated for treatment of glioblastoma in humans (Carpentier et al., 2016). Whereas various drug delivery approaches exploring the use of regular US contrast agents have shown some promise, their effectiveness is hampered by several issues. Being small, the magnitude of the biomechanical work that microbubbles of 1-8µm diameter can induce is relatively limited. In addition, as they are free flowing, they display limited contact with the endothelial wall, further reducing the level and range of any biomechanical effects (Kooiman et al., 2014). Furthermore, microbubbles are typically cleared from vascular compartments within 2–3 min and, finally, to produce sufficient biomechanical work and effect levels, microbubbles often need a high US intensity and as a consequence will potentially induce inertial cavitation, with ensuing potential safety issues.

More recently, a new microbubble concept, specifically designed to improve on the shortcomings of regular contrast microbubbles for targeted drug delivery, has been developed: acoustic cluster therapy (ACT) (Sontum et al., 2015; Healey et al., 2016). ACT addresses important deficiencies of microbubble contrast agents. In brief, ACT is defined as the co-administration of a drug together with a dispersion of microbubble/microdroplet clusters (PS101), followed by a two-step, local activation, and delivery-enhancement procedure targeted by ultrasound directed at the tumor. The microbubble in cluster acts as a vaporization seed, *i.e.,* the US activation forces the microbubble to oscillate which induces a liquid-to-gas phase shift of the microdroplet component and the formation of a large (22-μm diameter) bubbles. If the microbubble in the cluster was not present, the microdroplet would not phase shift at low acoustic MIs. The ACT bubbles produced by droplet vaporisation are designed to transiently lodge at the microvascular level of the vascular tree due to their size. The subsequent US enhancement step induces controlled volume oscillations that lead to enhanced local permeability of the vasculature, allowing for improved extravasation and distribution of drug into the tumor tissue extracellular matrix. The ACT bubbles, being 1,000 times larger (by volume) than regular contrast microbubbles, will induce in the range of three orders of magnitude greater biomechanical work. When lodged in the vascular compartment, the ACT bubbles display close contact with the endothelial wall over significant vessel segments; and remain for approximately 5–10 min. This allows for prolonged insonation and biomechanical work and these effects are induced using low intensity and low MI (<0.3) US. The concept represents a novel approach to targeted drug delivery that may improve significantly the efficacy of *e.g.,* current chemotherapy regimens.

Previously, ACT has been explored in combination with a range of drugs for treatment of several cancers, including; Abraxane^®^ (nab-paclitaxel) for treatment of prostate cancer (Van Wamel et al., 2016c) (Park, 2016) and paclitaxel for treatment of human pancreatic ductal carcinoma (PDAC) (Kotopoulis et al., 2017). In these earlier studies, a remarkable increase in the therapeutic efficacy over drug alone was observed when combined with the ACT procedure. In our work here we investigate the treatment of TNBC with liposomal doxorubicin (Doxil^®^) using two treatments of ACT. The therapeutic agent, Doxil, was chosen as it is considered a different drug class to the previously evaluated chemotherapeutic agents. Specifically, Doxil^®^ is a liposomal nanoparticle while the other evaluated nanoparticulate agent (Abraxane^®^) was a protein bound agent. This allows us to determine to what extent ACT is therapeutic agent agnostic. In addition, in a clinical trial using Doxil^®^ for treatment of metastatic breast cancer (O’Brien et al., 2004) Doxil^®^ showed reduced toxicity over doxorubicin but with no difference in efficacy. Here were evaluate if ACT can help improve the therapeutic efficacy of Doxil^®^, potentially adding an additional option for the treatment of metastatic breast cancer.

Furthermore, we perform a *post-hoc* analysis to investigate the relationship between ultrasound imaging contrast during the activation step and treatment efficacy as a biomarker for prediction and monitoring of therapeutic efficacy.

## Materials and Methods

### Cell Line and Tumor Model

The human TNBC cells MDA-MB-231.H-luc (American Type Culture Collection, Manassas, VA, USA, lot no. 8924081) were grown in Leibovitz’s L-15 cell culture medium supplemented with 10% fetal bovine serum in a humidified atmosphere of 5% CO_2_ at 37°C and passaged before renewal from frozen. Cells were regularly screened for mycoplasma by PCR using in-house primers.

Orthotopic tumors were established by injecting 3x10^6^ cells in 100 µl Matrigel:L-15 medium (1:4) into the thoracic mammary fat pad of 6-week old female athymic mice (ICR : NCr-Foxn1^nu^), bred in-house. During xenografting, mice were anesthetized using 2% isoflurane (Zoetis UK Ltd, UK) in oxygen. Mice were housed in groups of five in individually ventilated cages and allowed access to food and water *ad libitum*. All mice were treated in accordance with the local Animal Welfare and Ethical Review Board, the UK Home Office Animals Scientific Procedures Act 1986 and with the United Kingdom National Cancer Research Institute guidelines for the welfare of animals in cancer research (Workman et al., 2010).

The tumors were allowed to grow for up to 3 weeks and treatment was initiated when tumor volumes surpassed 180 (mm)^3^. The average (± SEM) starting volume was 209 ± 17 (mm)^3^ while the range was 183–241 (mm)^3^.

### Therapeutics

Clinical grade liposomal doxorubicin (DOX) (Caelyx^®^/Doxil^®^, Janssen Pharmaceutica NV, Belgium) was stored at 2–8 °C and freshly diluted for each treatment in 5% dextrose to a concentration of 1.5 mg/ml and administered as an intravenous (*i.v.*) bolus *via* a tail vein catheter. Mice were treated with DOX doses of 6 and/or 8 mg/kg matching literature values (Working et al., 1994).

PS101 was provided by Phoenix Solutions AS, Oslo, Norway (Sontum et al., 2015). PS101 was prepared by reconstituting commercially available microbubbles, Sonazoid™ (GE Healthcare), with a microdroplet emulsion of perfluoromethylcylopentane (PFMCP, F2 Chemicals Ltd., UK) microdroplets. The reconstituted PS101 formulation consists of a suspension of small microbubble/microdroplet conjugates (“clusters”) 6 x 10^7^ clusters/ml, with a median diameter of 5 µm (Sontum et al., 2015). The content of PFMCP, which is defined as the active pharmaceutical ingredient (API) in PS101, is 6.8 mg/ml. For administration of low doses, to allow for acceptable injection volumes, PS101 was diluted in 0.9% saline prior to administration.

### Treatment Protocol

Prior to each treatment anesthesia was induced by subcutaneous (*s.c*.) injection of fentanyl citrate +fluanisone (Hypnorm™, VetaPharma Ltd, Leeds, UK) and Midazolam (Hypnovel^®^, Roche Products Ltd, Welwyn Garden City, UK) (0.28:10:4.5 mg/kg). During treatments the mice were maintained on a mouse handling table (Vevo™, Fujifilm VisualSonics Inc., Toronto) and body temperature was controlled thermostatically, with vital signs carefully monitored. Following treatments mice were kept in a temperature-controlled recovery chamber until fully recovered.

**Figure 1:**
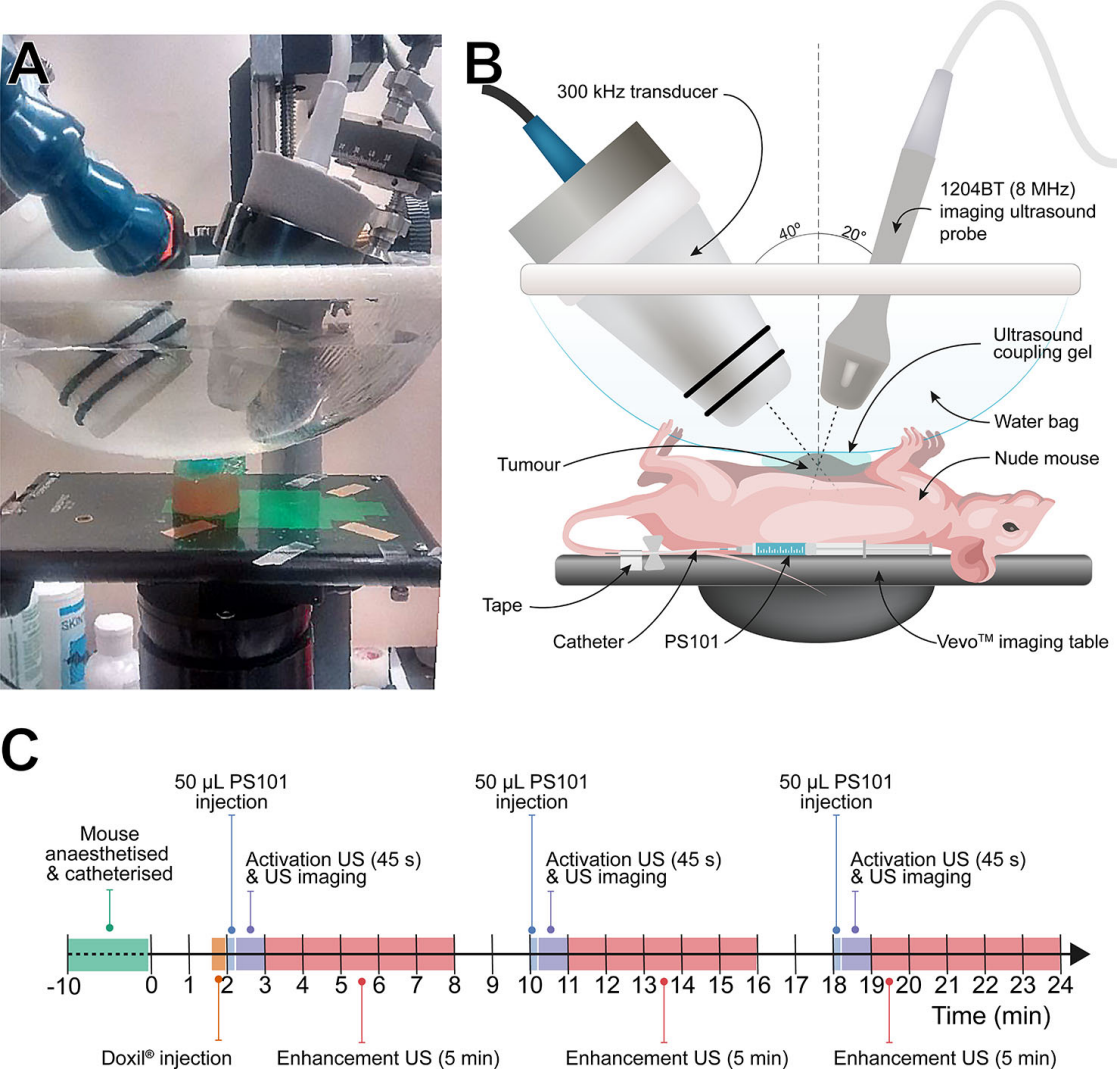
Overview of the experimental setup and experimental timeline. Panel **(A)**: Photograph of the experimental setup. A sample block has been positioned in place of the animal. Panel **(B)**: Schematic overview of the experimental setup with primary equipment labeled. Panel **(C)**: Timeline for each treatment cycle using acoustic cluster therapy (ACT) with Doxil^®^. Sham ultrasound was used for the Doxil^®^ group.

**Figure 1** shows a photograph (**Figure 1A**) and schematic explanation (**Figure 1B**) of the experimental setup used to image and treat the mice. The mice were placed in dorsal recumbency and ultrasound gel was applied to the tumor area only. An open polyethylene water bag was lowered until in contact with the ultrasound gel. **Figure 1C** shows the chronological order of each treatment cycle.

A clinical US diagnostic scanner, Aplio XG ultrasound scanner (Toshiba Medical Systems Corporation, Tochigi, Japan) combined with a 1204BT linear array ultrasound probe was used to both image the tumors and provide 45 s of “activation” ultrasound to phase-shift the PS101. The clinical system was set to an MI of 0.33 at a center frequency of 8 MHz at 20 fps. The clinical scanner operating in dual imaging mode permits visualization of the PS101 (Sonazoid component) inflow using non-linear contrast imaging, and ACT bubble formation (Healey et al., 2016; Van Wamel et al., 2016b) using B-mode imaging. At the end of the activation period, ultrasound output was switched to enhancement ultrasound applied *via* a custom made 300 kHz, 55 mm diameter, single element focused transducer (Imasonic SAS, Voray-sur-l’Ognon, France) for 5 min at an MI of 0.15 with two cycles of excitation every 125 ms. The 300 kHz ultrasound transducer was driven by an Analogic 2045 polynomial waveform synthesizer (Analogic Corp, USA) amplified by a pulsed radio frequency (RF) amplifier, BT00500 (Tomco Technologies, Australia) power amplifier. The acoustic conditions were chosen based on *a-priori in-vitro* and *in-vivo* experiments (Healey et al., 2016; Van Wamel et al., 2016a)

During activation, both B-mode and contrast-mode images were acquired for correlation with therapeutic response. The two transducers were fixed and aligned in relation to each other so that the imaging focus of the diagnostic array and the acoustic focus of 300 kHz transducer coincided without interfering with the acoustic propagation of the other transducer. Specifically, the 1204BT array was placed at 20° off the vertical axis and the 300 kHz array −40° off the vertical axis. Once the mouse was in place, both ultrasound sources where lowered so that the acoustic foci aligned with the center of the tumor, and the transducers’ front surfaces were within the water bag. The acoustic pressures of the 300 kHz transducer were calibrated *a priori in situ* using a 200-µm lipstick hydrophone (HGL-200, Onda Corporation, Sunnyvale, CA, USA). The hydrophone was spot calibrated in-house at 300 kHz by comparing to a fully calibrated needle hydrophone (1-mm, Precision Acoustics Ltd, Dorchester, Dorset, UK). The scanner’s on-screen values were used for the 8-MHz transducer.

For PS101 and drug delivery a catheter was assembled by combining a winged infusion set, Surflo^®^ 27G butterfly needle (Terumo Europe, Leuven, Belgium), a 70 mm length of polyethylene tubing, 0.4 mm i.d. (Biochrom Ltd, Cambridge, UK) and a 27G, 0.5” hypodermic needle. It was validated *a priori* using established methods (Sontum et al., 2015), that this exact injection procedure did not affect the size distribution or activation potential of the PS101 (**Supplemental Figure 1**, **Supplemental Table 3**). The catheter, primed with a 0.9% saline solution, was inserted into the lateral tail vein of the mouse and patency checked by injecting a small volume of saline solution <5 µl. The hub of the cannula was then filled with 0.9% saline and closed with a cap and taped to the animal’s tail with surgical tape, which resulted in a “dead space” of 10 µl to be accounted for in subsequent injections. Just prior to each new injection, PS101 was freshly drawn up into a 1 ml syringe and 60 µl (50 µl effective dose, plus 10 µl to allow for dead space) was injected intravenously into the animal’s lateral tail vein *via* the preplaced catheter. Three injections of PS101 followed by activation and enhancement ultrasound were performed for each treatment cycle.

### Treatment Groups

**Table 1** summarizes the treatment groups evaluated in this study. Animals were randomized into cohorts of 8–10 mice randomly, depending on when the tumors reached the required starting volume. Group 1 was only PS101 followed by activation and enhancement ultrasound. Group 2, treated with Doxil^®^ only, employed a reduced Doxil^®^ dose during the second treatment cycle to match the lower doses employed in group 3, ACT with Doxil^®^, where dose was lowered during the first treatment cycle to compensate for apparent toxicity encountered when treating the first animals with a dose of 8 mg/kg in the first treatment cycle. This toxicity manifested as a failure to recover from the anesthetic procedure. Hence the dose reduced to 6 mg/kg was used in the ACT with Doxil^®^ group and maintained for both treatment days.

**Table 1:** Summary of the treatment groups: number of mice, name, and Doxil^®^ and acoustic cluster therapy (ACT) doses; t_1_ and t_2_ indicate the doses at the fist (day 0) and second (day 21) treatment cycle.

Group	Name	Number of animals	Treatment doses		US exposure
			Doxil^®^ (mg/kg) t_1_→t_2_ (total)	ACT 3×(ml/kg) t1→t2	
1	PS101+US	9	–	2.00→2.00	✓
2	Doxil^®^	10	8→6 [14]	–	✕
3	ACT with Doxil^®^	8	6→6 [12]	2.00→2.00	✓

### Disease Development Evaluation

Animal health status was monitored daily. Tumor volumes were obtained *via* caliper measurement four to five times a week up to 175 days from study start. Tumor volumes were calculated using the ellipsoid equation $\frac{4}{3}\pi {\left(\frac{a}{4}+\frac{b}{4}\right)}^{3}$.

Tumor size is reported as fold increase normalized to the day of the first treatment, *i.e.,* day 0. Values were linearly interpolated to single day values for graphing purposes.

Following the 3Rs of ethical research and EU directives (Directive 2010/63/EU, 2010) a drug + US only group was not included in the study as the US exposure levels are well below those which might cause bioeffects (Miller et al., 2008; Nelson et al., 2009). Similarly, Sonazoid™ and saline in the absence of US are not expected to affect tumor growth, and such groups were not included.

Mice that showed no visible sign of the tumors at the end of the study (day 175) were considered complete responders while mice that were sacrificed prior to the end of the study were considered non-responders. To minimize experimental bias and animal suffering, a score sheet was used to determine when to sacrifice an animal based on ulceration and tumor size (**Supplemental Table 1**)

### Response Assessment

#### Contrast Enhancement Ranking

Contrast enhancement ranking (*i.e.,* Imaging rank) was determined from the ultrasound images recorded during activation following post-processing in MATLAB 2014a (MathWorks, Massachusetts, NA, USA). Specifically, an ROI is manually defined within the tumor core in the contrast image. The average image intensity for frame 1 (reference image pre-PS101 injection) and frame 300 (contrast enhanced image 15 s after PS101 injection) and the difference between frame 1 and frame 300, was calculated. This was repeated for all three PS101 injections on day 0 and day 21. The average of these six values (three from each treatment day over two treatment days) was used as the contrast metric, and determined the imaging rank. These values were then used to produce a ranked score of contrast enhancement by sorting them in increasing order of contrast enhancement where a larger average contrast value is a higher imaging rank. The attained measured values were also used to evaluate if there were any changes in tumor perfusion over the three sequential treatments on either of the two treatment days.

### Therapy Response Ranking

Non-responding animals, *i.e.,* animals that were sacrificed prior to 175 days, were ranked by survival time (shorter survival ranked lower). Complete responders were ranked by the day the tumor volume reached zero and stayed at zero *i.e.,* the shorter the time to reach zero tumor volume is a higher therapy rank. Both the contrast enhancement and therapy response ranking were performed by observers who were blinded to the other ranking.

### Statistical Analysis

Results for average tumor normalized volume are expressed as mean ± standard error. Survival was compared using a Log-rank (Mantel-Cox) test between two groups. Contrast enhancement for comparing perfusion changes was evaluated using a simple linear regression. Therapy and imaging rank correlation was performed using nonparametric Pearsons correlation. A contingency table was used to compare the number of complete responders *via* Fishers exact test. A p-value less than 0.05 was considered statistically significant. All statistical analysis was performed in Prism 8.3.0 (GraphPad Software Inc, San Diego, CA, USA).

## Results

### Tumor Growth and Development

**Figure 2:**
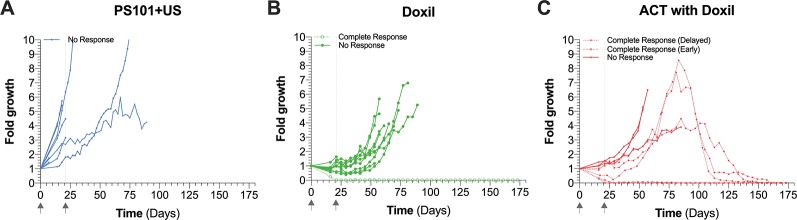
Normalized tumor growth as a function of time. Each panel shows all the mice for the respective groups. Mice unable to survive the complete treatment period are considered non-responders. Gray arrows indicate the two treatment time points. Panel **(A)** shows the growth curves of the mice treated with PS101+US. Panel **(B)** shows the growth curves of the mice treated with Doxil^®^. Panel **(C)** shows the response of the mice treated with acoustic cluster therapy (ACT) with Doxil^®^.

**Figure 2** shows the normalized tumor growth as a function of time for all three groups. For ease of visualization, markers are plotted every 4 days. **Supplemental Figure 2** shows the exact tumor volumes as a function of time.

When treated with PS101+US (**Figure 2A**) no therapeutic response was observed, as expected. The tumor growth curves in this group show a bimodal distribution; in one population 2 out of 9 mice (22%) had a two-fold tumor growth by day 21. The second population (78%) showed a rapid growth in the range of 2 to 6-fold growth over the first 21 days. All mice in the first population were able to survive past 21 days in contrast to only one mouse in the second population.

The mice treated with Doxil^®^ (**Figure 2B**) showed a marked improvement over the PS101+US group with all mice surviving both treatment cycles. All mice showed either tumor growth stagnation or regression over the first 21 days. Once again, there was an inhomogeneous or bimodal distribution. One mouse (10%) showed complete regression/response by day 21; the remaining population (90%) showed tumor growth/recovery within 4 weeks after the last treatment (by day 49).

Treating mice using ACT with Doxil^®^ showed a marked improvement over Doxil^®^ alone (**Figure 2C**). Similar to both the PS101+US and Doxil^®^ groups, there was an inhomogeneous population; in this instance three populations could be discerned. By day 21, three mice (38%; mice 4, 5, and 6 *c.f.*, **Supplemental Figure 3**) showed tumor regression. Two of the three mice (mice 4 and 5 *c.f.*, **Supplemental Figure 3**) stayed in complete remission until the end of the study (first population). Three mice (mice 6, 7, and 8 *c.f.*, **Supplemental Figure 3**) showed tumor re-growth after the second treatment cycle but presented a delayed response resulting in complete remission starting 70 ± 2 days after the last treatment cycle (*i.e.,* day 91) (second population). The third population (3 mice; 38%; mice 1, 2, and 3 *c.f.*, **Supplemental Figure 3**) showed continuous tumor re-growth and did not survive the entire study period.

**Figure 3:**
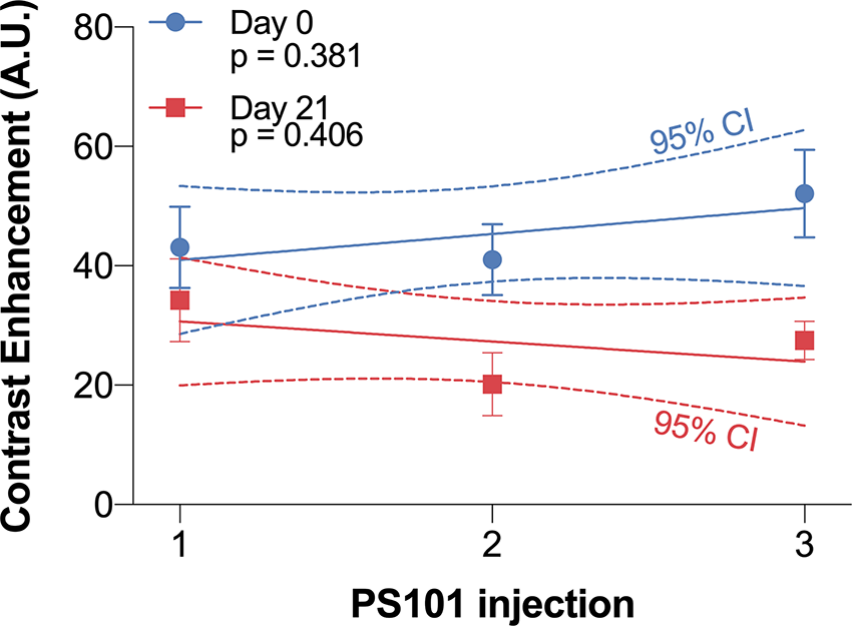
Contrast enhancement difference 15 s post-PS101 injection for each of the three injections for day 0 (1^st^ treatment) and day 21 (2^nd^ treatment). The linear regression slope between the three sequential injections was not significantly different from zero on day 0 (p = 0.381) or day 21 (p = 0.406).

**Figure 3** compares the perfusion of the ACT with Doxil^®^ tumors over the three sequential injections for both treatment days. The linear regression was not significantly different from zero for either of the treatment days indicating similar tumor perfusion in all three sequential injections.

### Survival

#### Median Overall Survival

**Figure 4:**
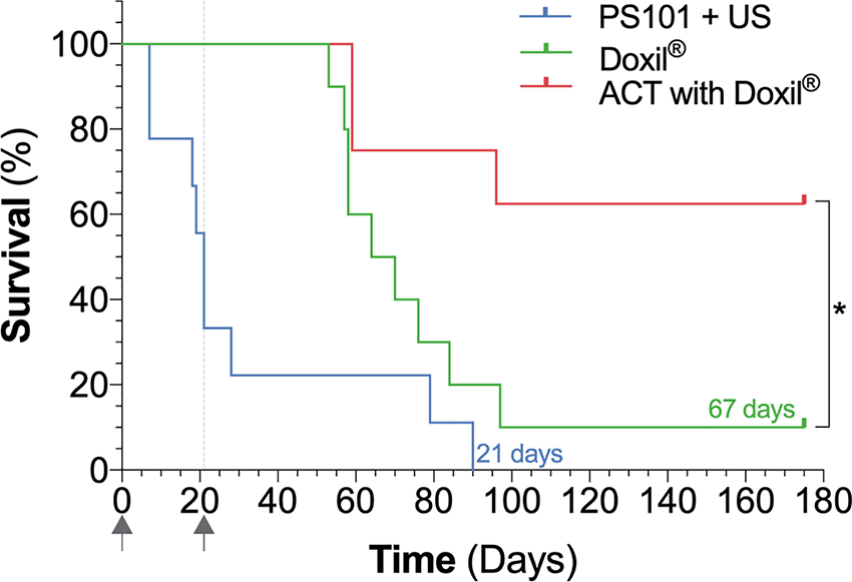
Survival curves of the three groups evaluated in the study. *p = 0.02.

**Figure 4** shows the survival curves for the animals in the three study groups. PS101+US resulted in a median overall survival of 21 days. Treating with Doxil^®^ improved overall survival significantly to 67 days (p = 0.04 *vs.* PS101+US) while treating using ACT with Doxil^®^ further improved survival (p = 0.02 *vs.* Doxil^®^, p = 0.0004 *vs.* PS101+US).

#### Complete Responders

Both the Doxil^®^ and ACT with Doxil^®^ group had complete responders, *i.e.,* mice that showed no signs of tumor burden and survived the complete study period of 175 days. There were no survivors for the PS101+US group. The Doxil^®^ group had a single complete responder (10%), while the ACT with Doxil^®^ group had five complete responders (63%) clearly indicating the benefit of ACT; this difference was significant (p = 0.03).

### Imaging Biomarkers of Therapeutic Response

**Figure 5:**
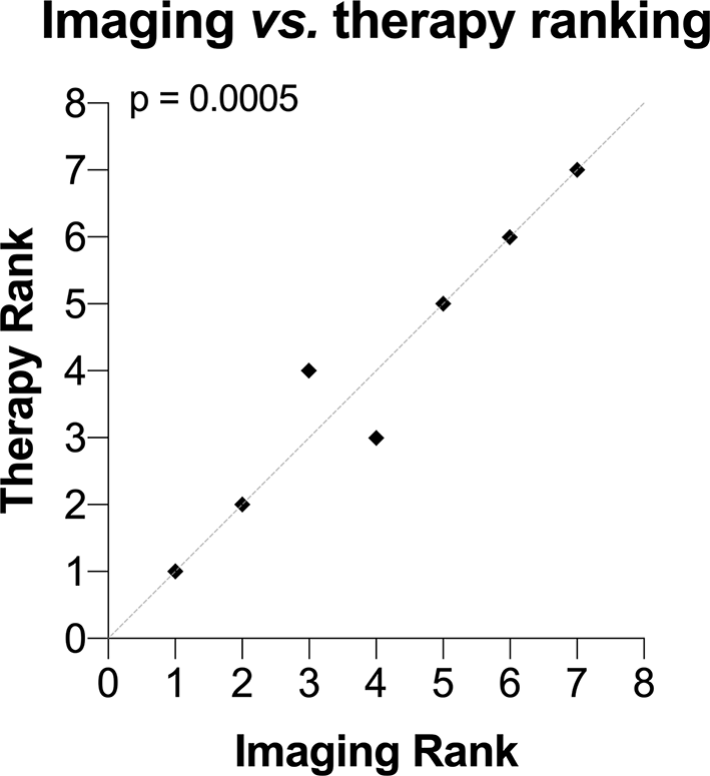
Correlation between ultrasound imaging contrast rank and therapy rank. A significant correlation between the imaging and therapy rank was observed (p = 0.0005). The dashed gray line is the line of identity.

**Figure 5** shows the correlation between the imaging and therapy rankings. A significant positive correlation was observed (p = 0.0005, r = 0.96, Pearson r) indicating that non-linear contrast is a predictor of therapeutic outcome when using ACT with Doxil^®^. The therapy ranking of each mouse is shown in **Supplemental Table 2**.

**Figure 6:**
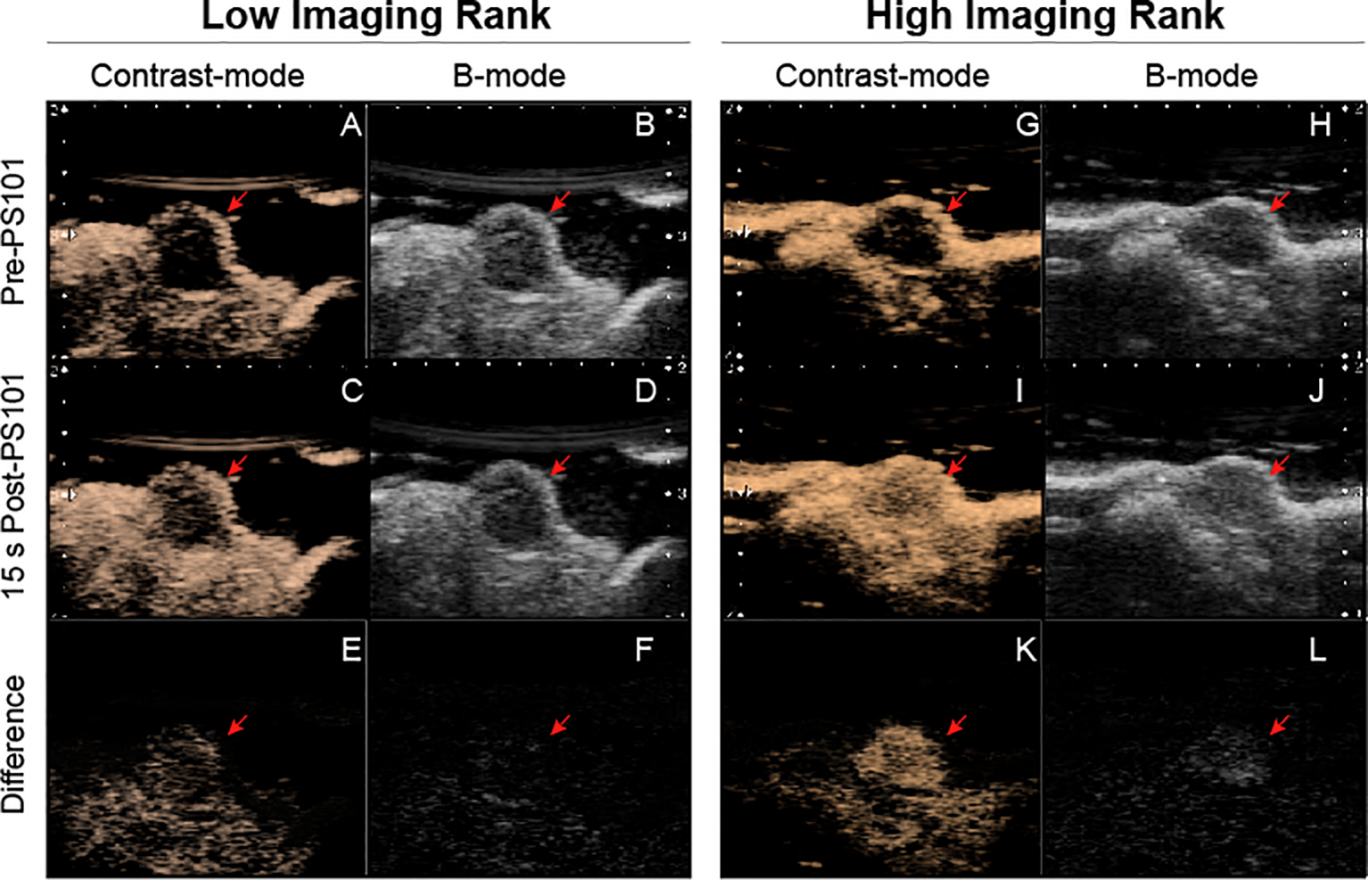
Ultrasound images of two acoustic cluster therapy (ACT) with Doxil^®^ mice just before and during the activation ultrasound procedure. The left panel (frames **A–F**) shows a low contrast image-ranked tumor and the right panel (frames **G–L**) shows a high contrast image-ranked tumor. The first row (frames **A**, **B**, **G**, and **H**) are prior to PS101 injection. The second row (frames **C**, **D**, **I**, and **J**) are 15 s post-PS101 injection, while activation ultrasound is being applied. The third row (frames **E**, **F**, **K**, and **L**) shows the difference between first and second row. A pronounced difference is observable between the low image-ranked tumor and high image-ranked tumor in both the contrast-mode and B-mode images.

**Figure 6** shows example images of a low and a high imaging contrast ranked mouse from the ACT with Doxil^®^ group. The sepia toned frames are example non-linear imaging mode frames that help visualize tumor perfusion. The last row (row 3), of images are subtractions of the pre-PS101 images (row 1) from the 15 s post-PS101 images (row 2). This subtraction removes the tissue harmonic component and emphasizes the Sonazoid component. In the two illustrated examples, pre-PS101 (**Figure 6**, first row), the tumors (red arrows) appear as a hypoechoic region in both B-mode and contrast-mode images and little difference can be seen between the two. The contrast observed is due to the tissue harmonic imaging. In the low imaging contrast ranked mouse (*Animal 2* in **Supplemental Figure 3** and **Supplemental Table 2**) very little enhancement can be seen when comparing pre-PS101 injection to 15 s post-PS101 injection, both in B-mode and non-linear contrast mode images, *i.e.,* the tumor remains hypoechoic (*c.f.,* **Figures 6A, B** *vs.* **Figures 6C, D**). This minimal change in image brightness can be clearly observed in the difference frames, both for contrast-mode and B-mode (**Figures 6E, F**). These frames only show the presence of microbubbles, as the tissue harmonic contrast component is removed *via* subtraction.

In the high imaging contrast ranked mouse (*Animal 7* in **Supplemental Figure 3** and **Supplemental Table 2**) 15 s post-PS101 injection a contrast enhancement can be observed in both B-mode and contrast-mode resulting in the tumor being visualized going from hypo-echoic to hyper echoic (*c.f.,* **Figures 6G, H** *vs.* **Figures 6I, L**). The difference frames (**Figures 6K, L**) clearly show the tumor noticeably bright in both the contrast-mode and B-mode indicating that this tumor is better perfused than the low imaging rank tumor.

## Discussion

The use of ACT with Doxil^®^ shows a significant improvement in therapeutic response *versus* Doxil^®^ alone, indicating the potential of ACT to work synergistically with a liposomal nanoparticle drug formulation. This study shows that ACT with Doxil^®^ can significantly improve the percentage of complete responders and extends overall survival. In addition, our results show that microbubble contrast enhancement of the tumor can be used as a therapeutic biomarker predicating the efficacy of ACT with Doxil, where more contrast enhancement indicates a potential for better treatment.

### Tumor Model

All groups in this study showed an inhomogeneous tumor growth behavior in response to therapy, similar to that observed in clinical practice which is also given as a key factor behind the difficulty in successfully treating TNBC (Bianchini et al., 2016). In clinical disease progression, metastatic spread is also a significant reason for poor prognosis, Metastatic spread was, however, not evaluated in this study, the reason being that due to the high toxicity of treating with Doxil^®^, the addition of a whole body imaging step to detect metastasis (such as bioluminescence imagine) may have increased the stress on the mice, potentially introducing early dropouts. Metastatic spread should, however, be evaluated in future studies to determine if there are correlations with overall survival and primary tumor size when treating using ACT with Doxil^®^.

### Tumor Growth Inhibition and Survival

As expected, PS101+US showed the lowest overall survival indicating that PS101+US had little to no effect on tumor growth. To verify this an additional control group that received no treatment would have been ideal but was avoided to reduce the use of animals and unnecessary stress (Directive 2010/63/EU, 2010).

Doxil^®^ showed a significant effect with the majority of animals having tumor regression, despite this, all but one mouse showed tumor re-growth after the two cycles of treatment. Transferring this to a clinical scenario, this would indicate that a patient may need many more treatment cycles or continuous therapy for an improved outcome.

When performing ACT with Doxil^®^, a synergistic effect was observed greatly improving the therapeutic efficacy of Doxil^®^. Some tumors showed rapid regression after a single treatment cycle. One of these mice (**Supplemental Figure 3**, mouse 6) showed re-growth once treatment was stopped. This single mouse showed a delayed response, and by the end of the treatment, no tumor could be detected. Furthermore, after a few weeks, the majority of mice (63%) had no observable tumors over 154 days after the last treatment. This indicated a significant improvement in response rate and therapeutic benefit, especially when compared to Doxil^®^ alone. If such results could directly translate to the clinical response, this would indicate that patients would need fewer treatment cycles, resulting in less toxicity, with an improved therapeutic outcome.

When comparing the survival curves, ACT with Doxil^®^ was significantly better than Doxil^®^. While the Doxil^®^ group had a median overall survival of 67 days, due to the high curative efficacy of ACT with Doxil^®^ it was not possible to calculate a representative median survival, further emphasizing the marked improvement over Doxil^®^.

PS101+US had no effect on tumor growth or survival by itself, whereas when performed with Doxil^®^ a significant improvement was observed. This may imply that the mechanical action of PS101+US is able to enhance the efficacy of Doxil^®^ by either increasing drug delivery or further sensitizing tissue; however, more research should be performed to determine the mechanisms of action behind the *in-vivo* synergy of ACT with therapeutic agents.

### Imaging Biomarkers of Therapeutic Response

The correlation between the ultrasound image contrast and therapeutic outcome indicates that the more perfused a tumor is the better the treatment efficacy. This may indicate that higher perfusion allows more PS101 and/or more drugs to enter the tumor volume and may be a requisite for successful ACT based therapy. This indicates that tumor perfusion may be used as a therapeutic biomarker or predictor of efficacy. With the need for personalized medicine being the next evolution of current treatment strategies this points toward ACT based therapies being an optimal choice for the next generation of therapeutics (Tran et al., 2018).

Comparing clinical findings for various breast cancer types, tumours of patients with TNBC show the largest amount of microbubble perfusion when using contrast enhanced ultrasound indicating the potential for ACT in treating patients with TNBC (Masumoto et al., 2016).

The primary limitations of this data set are the limited number of animals (n = 8), the limited proportion of the volume of each tumour (only a single imaging plane), that were studied. Future work should aim to determine the sensitivity and specificity of such imaging biomarkers using sufficient animals for adequately powered estimates, as well as characterize PS101 uptake in the entire tumor volume.

### Limitations and Future Work

While this study provides strong evidence that ACT with Doxil^®^ can enhance the therapeutic efficacy versus Doxil^®^ alone, there are several limitations that should be addressed in future studies.

The underlying mechanisms of this enhanced therapeutic effect were not evaluated within this study. While it may be assumed that this is due to increased delivery of the chemotherapeutic agent or changing the release profile this has not been directly proven within this model. Nevertheless, previous work has demonstrated that ACT is able to enhance the deposition of large dyes molecules (2.5 nm diameter) into tissue *in vivo* (Van Wamel et al., 2016b) indicating that this may be a contributory mechanism. The mechanism underlying the delayed therapeutic response is, however, as yet not clearly understood.

In this study, three sequential injections of PS101 were performed, spaced only 6 min apart. As the *in-vivo* lifetime of PS101 can be longer than this time period, this allows the assumption that each subsequent injection may have residual un-activated PS101, increasing the attenuation with each injection. Consequently, further work should be performed to optimize the PS101 dose and acoustic conditions to account for this potential phenomenon.

Vascular shut down due to ultrasound and microbubble treatment has been reported at high mechanical indices [*e.g.,* MI 1.6 (Goertz et al., 2012)]. In our study here, no vascular shutdown was observed either after any of the three PS101 injections or on any of the treatment days. This indicates that vascular shut down may not be part of the mechanisms of action of ACT. This also supports the use of the three back-to-back injection of PS101 as the clusters will still be able to perfuse through the tumor.

To verify that the improved efficacy seen with ACT, over Doxil^®^ alone, is due to the large activated ACT bubbles, not just the Sonazoid component, future work should include a Sonazoid control group.

Although the off-target toxicity/safety was not directly evaluated in this study it remains a key point of interest for such a targeted drug delivery mechanism. Extensive studies have been performed in several species and determined that there are minimal toxicities induced by ACT and, when they exist, they are transient and recoverable (Myhre et al., 2016; Bush et al., 2019).

While ultrasound and microbubbles have been used to enhance the treatment efficacy for TNBC (Bai et al., 2019; Jing et al., 2019; Song et al., 2019), this has not been previously explored with ACT and to our current knowledge, no other study has shown such a marked improvement in tumor regression, significantly improved survival and number of complete responders, and theranostic potential.

## Conclusion

ACT significantly improves the response to treatment with Doxil^®^ of human triple negative breast cancer in mice, as measured by tumor size and overall survival, with 63% of tumors entering complete regression with ACT versus 10% with Doxil^®^ alone. ACT has potential theranostic attributes and ultrasound contrast enhancement during or before ACT treatment may be employed as a biomarker of therapeutic response and, potentially, for patient stratification in clinical management.

## Data Availability Statement

The datasets generated from this study can be found in the public repository hosted by “LabArchives” (https://mynotebook.labarchives.com/share_attachment/My%2520Notebook/MjMuNDAwMDAwMDAwMDAwMDAyfDQ5MDQxMS8xOC02L1RyZWVOb2RlLzMwMzgyMDMyMTV8NTkuNA==).

## Ethics Statement

The animal study was reviewed and approved by The ICR Animal Welfare & Ethical Review Body.

## Author Contributions

NB, AH, AS, GB, VK, SE, PS, SvK, AW, CD, and JB contributed to the conception and design of the study. NB, AH, and GB performed the experiments. NB, AH, SpK, SvK, PS, and JB all co-wrote the manuscript. All authors contributed to the manuscript revision, read and approved the submitted manuscript.

## Funding

This research was funded by Phoenix Solutions AS, partially through Research Council of Norway grant # 228604.

## Conflict of Interest

AH, SpK, SvK, and PS were employed by the company Phoenix Solutions AS at the time of manuscript submission. NB, AH, SvK, PS, and AV are shareholders of Phoenix Solutions AS at the time of submission. The authors declare that this study received funding from Phoenix Solutions AS. The funder had the following involvement with the study: study design, data analysis, decision to publish and preparation of the manuscript.

The remaining authors declare that the research was conducted in the absence of any commercial or financial relationships that could be construed as a potential conflict of interest.

The two reviewers declared their involvement as co-editors in the Research Topic, and confirm the absence of any other collaboration.
