# Supplementary material for: Theranostic Attributes of Acoustic Cluster Therapy and Its Use for Enhancing the Effectiveness of Liposomal Doxorubicin Treatment of Human Triple Negative Breast Cancer in Mice
Source: Front Pharmacol. 2020 Feb 20;11:75. doi: 10.3389/fphar.2020.00075 (PMC7044119; doi:10.3389/fphar.2020.00075)
Supplement: Supplementary file 1 [file DataSheet_1.docx]

Supplementary Material

| **Section** | **Lesion** | **Score** | **Notes** | |
| --- | --- | --- | --- | --- |
| **A** | Dry scab/crust, coherent skin covering | 0.5 | Monitor all lesions at least twice weekly.  On a separate sheet record date, exp. no., box no., mouse no., score, initial | |
|  | Small focal skin breakdown (<1 mm) | 1 |  |  |
|  | Chronically wet/weeping scab/crust or sold yellow matter exposed | 2 |  |  |
|  | Acute burst releasing fluid/pus or acute split at border | 2 |  |  |
|  | Bleeding on raw tissue exposed or white basal later | 3 |  |  |
| **B** | **Behavior** | **Score** | If you have scored 3 in sections A or B cull the same day. | |
|  | Normal | 0 |  |  |
|  | Recurrent grooming of tumour site or abnormal gai not impeding locomotion | 1 |  |  |
|  | Recurrent scratching/biting of tumour site | 2 |  |  |
|  | Impeded movement or function | 3 |  |  |
| **C** | **Size/progression of lesion** |  | **Scab** | **Open Ulceration** |
|  | Shrinking |  | 0 | 0 |
|  | Static |  | 0 | 1 |
|  | 2–3 mm growing |  | 0.5 | 2 |
|  | 3–5 mm growing |  | 0.5 | 3 |
|  | 5+ mm growing |  | 1 | 4 |
|  | **Size/progression of tumour** |  |  | |
|  | Shrinking |  | 0 | 0 |
|  | Static |  | 0 | 1 |
|  | 10–12 mm growing |  | 1 | 2 |
|  | 12–14 mm growing |  | 2 | 4 |
|  | 14 mm growing Mean of D1/D2 or D1 or D2 is 17 mm) |  | 3 | 6 |

**Supplemental Table 1:** Tumour ulceration score sheet. If total for section C or A+B+C combined is greater than or equal to 6, then cull within 24 hours. If 4-5, monitor daily and cull within 1 week if no improvement

| **Animal Number** | **Survival** | **Therapy Index** | **Contrast Value [A.U.]** | **Imaging Index** |
| --- | --- | --- | --- | --- |
| 1 | Culled day 48 | 1 | 25 | 1 |
| 2 | Culled day 48 | 1 | 25 | 1 |
| 3 | Culled day 85 | 2 | 30 | 2 |
| 4 | 0 vol. day 74 | 6 | 49 | 6 |
| 5 | 0 vol. day 10 | 7 | 70 | 7 |
| 6 | 0 vol. day 160 | 5 | 44 | 5 |
| 7 | 0 vol. day 175 | 4 | 31 | 3 |
| 8 | 0 vol. day 153 | 3 | 34 | 4 |

**Supplemental Table 2:** Therapy and Imaging ranking scores for each mouse in the ACT with Doxil group.

| **Sample** | **Description** | **Volume peak  (x10^-3^ %)** | **Diameter at peak  (µm)** | **Half-time (s)** |
| --- | --- | --- | --- | --- |
| 1 | In vial, standard sample | 1.03 | 50 | 51.5 |
| 2 | Through 30G needle | 1.16 | 50 | 48.5 |
| 3 | In vial, standard sample | 1.21 | 50 | 50 |

**Supplemental Table 3:** Sonometry results evaluating the effect of injecting PS101 through a 30G needle. No change in volume peak, diameter at peak and half-time is observed indicated using a 30G needle does not affect phase shift behavior or concentration PS101 and subsequent ACT bubbles.

**Supplemental Figure 1:** Coulter counter results evaluating the effect of injecting PS101 through a 27G needle. No change in the size distribution and particle count is observed indicating that the PS101 clusters stay intact when passing through a 27G needle.

**Supplemental Figure 2:** Tumour growth as function of time. Each panel shows all the mice for the respective groups. Grey arrows indicate the two treatment timepoints. **Panel A** shows the growth curves of the mice treated with PS101+US. **Panel B** shows the growth curves of the mice treated with Doxil^®^. **Panel C** shows the response of the mice treated with ACT with Doxil^®^.

**Supplemental Figure 3:** Normalised tumour growth curves for the ACT with Doxil^®^ group. Each colour coordinated number corresponds to the animal number seen in **Supplemental Table 2**.
